# Tenofovir disoproxil fumarate directly ameliorates liver fibrosis by inducing hepatic stellate cell apoptosis via downregulation of PI3K/Akt/mTOR signaling pathway

**DOI:** 10.1371/journal.pone.0261067

**Published:** 2021-12-08

**Authors:** Sung Won Lee, Sung Min Kim, Wonhee Hur, Byung-Yoon Kang, Hae Lim Lee, Heechul Nam, Sun Hong Yoo, Pil Soo Sung, Jung Hyun Kwon, Jeong Won Jang, Seong-Jun Kim, Seung Kew Yoon

**Affiliations:** 1 Department of Biomedicine & Health Sciences, The Catholic University Liver Research Centre, POSTECH-Catholic Biomedical Engineering Institute, The Catholic University of Korea, Seoul, Republic of Korea; 2 Department of Internal Medicine, College of Medicine, The Catholic University of Korea, Seoul, Republic of Korea; 3 Korea Research Institute of Chemical Technology, Daejeon, Republic of Korea; National Institutes of Health, UNITED STATES

## Abstract

**Background:**

Antifibrotic agent for the treatment of liver fibrosis has not been developed so far. Long term treatment of chronic hepatitis B patients with antiviral drugs tenofovir disoproxil fumarate (TDF) and entecavir (ETV) results in the regression of liver fibrosis, but the underlying mechanism has not been clarified. Therefore, we aimed to investigate the direct impact of TDF and ETV on liver fibrosis.

**Methods:**

Activated hepatic stellate cell (HSC) cell lines were used to evaluate the effects of TDF and ETV. After treatment with each antiviral agent, cell viability, morphology, apoptotic features, autophagy and antifibrosis signalling pathways were examined. Then, collagen deposition, fibrosis markers and activated HSCs were measured in liver tissues of the liver fibrosis model mice.

**Results:**

After TDF treatment, the viabilities of LX2 and HSC-T6 cells were decreased, and the cells exhibited apoptotic features, but ETV did not induce these effects. Cleavage of PARP and Caspase-3 and the inhibition of the antiapoptotic gene Bcl-xl indicated activated HSC apoptosis following TDF treatment. TDF simultaneously increased autophagy, which also regulated apoptosis through crosstalk. TDF inactivated the PI3K/Akt/mTOR signalling pathway, which was associated with the activation of both apoptosis and autophagy. In the liver fibrosis mouse model, the fibrotic area and activated HSC markers were decreased by TDF but not ETV treatment. Additionally, apoptotic cells were concentrated in the periportal fibrotic area after TDF treatment, which indicated the specific antifibrotic effect of TDF.

**Conclusions:**

TDF directly ameliorates liver fibrosis by downregulating the PI3K/Akt/mTOR signalling pathway, which results in the apoptosis of activated HSCs. The antifibrotic effects of TDF indicate that it may be a therapeutic agent for the treatment of liver fibrosis.

## Introduction

Liver fibrosis is a pathological consequence of the repair mechanism associated with chronic liver injury induced by various causes, such as hepatitis B virus (HBV), hepatitis C virus (HCV), excessive alcohol intake, and non-alcoholic steatohepatitis (NASH). Progressive liver fibrosis eventually leads to liver cirrhosis, which is one of the major causes of morbidity and mortality worldwide [1]. The burden of liver cirrhosis has constantly increased over the past thirty years from 676,000 deaths in 1980 to over one million deaths in 2010, but antifibrotic agents for the treatment of liver fibrosis have not been developed thus far [2].

To date, various underlying molecular and cellular mechanisms of liver fibrosis have been elucidated, but these mechanisms contribute to hepatic fibrogenesis through a complex network. Current treatment strategies to inhibit or cure hepatic fibrosis include eliminating the cause of chronic liver disease, controlling inflammation, inhibiting excessive extracellular matrix (ECM) production and regulating cellular viabilities.

Hepatic stellate cells (HSCs) play a crucial role in the fibrogenic process, as their activation induces the synthesis of collagen and tissue inhibitors of metalloproteinase (TIMPs), consequently resulting in the excessive production of extracellular matrix in the liver [3]. During the early stages of HSC activation, growth factors such as TGF-β activate Smad-dependent or Smad-independent pathways [4], and platelet-derived growth factor (PDGF), a highly potent chemoattractant of HSCs, stimulates the phosphoinositide 3-kinase (PI3K)/protein kinase B (Akt)/mammalian target of rapamycin (mTOR) signalling pathway [5–7]. The PI3K/Akt/mTOR pathway is physiologically involved in various processes, such as cell proliferation, differentiation, autophagy and apoptosis, but its activation has been reported to be associated with fibrogenesis [8–11].

Tenofovir disoproxil fumarate (TDF) is currently the most widely used antiviral agent for the treatment of chronic hepatitis B (CHB). TDF is a nucleotide reverse transcriptase inhibitor and a prodrug of tenofovir that suppresses HBV replication in hepatocytes. However, recent clinical studies have revealed that treatment with TDF results in not only in viral suppression but also the regression of liver fibrosis. Marcellin et al. reported that after 5 years of treatment with TDF, 96% of patients exhibited either improved or unchanged fibrosis and that 73% of patients with established cirrhosis showed improvements of two stages or greater [12]. Long-term treatment with other antiviral drugs has also been reported to improve liver fibrosis through the removal or suppression of causative agents. However, Papatheodoridis et al. recently suggested in their European multicentre study that TDF was even superior to entecavir (ETV), another highly potent antiviral drug, in reversing cirrhosis [13–15].

Therefore, the aims of this study were to investigate and compare the direct antifibrotic effects of the antiviral agents TDF and ETV and to elucidate the underlying mechanisms associated with the amelioration of liver fibrosis.

## Materials and methods

### Animal studies

Eight-week-old C57BL6 male mice were purchased from Orient Bio Inc. (Seoul, Korea) and housed at 22°C ± 5°C with a 12 h light/dark cycle. Thioacetamide (TAA, Sigma-Aldrich, St Louis, MO, USA) was administered to induce fibrosis. The mice were randomly divided into four groups (5 mice in each group): normal, TAA (only TAA injection), ETV (TAA injection and ETV 0.2 mg/kg/day), and TDF (TAA injection and TDF 100 mg/kg/day). The TAA group was administered 150 mg/kg TAA intraperitoneally 3 times a week for 10 weeks. All antiviral drugs were dissolved in drinking water containing 0.5% carboxymethylcellulose (Sigma-Aldrich) and were administered daily by oral gavage for 10 weeks. The mice were sacrificed and their livers harvested. Isolation of primary hepatocytes and stellate cells (HSCs) from mouse liver was performed as previously described [16]. All procedures were performed in accordance with the Laboratory Animals Welfare Act, the Guide for the Care and Use of Laboratory Animals and the Guidelines and Policies for Rodent Experiments provided by the Institutional Animal Care and Use Committee (IACUC) of the Catholic University of Korea (approval number: CUMS-2018-0229-02) and the National Institutes of Health guide for the care and use of Laboratory animals (NIH Publications No. 8023, revised 1978). Serum alanine aminotransferase (ALT) and creatinine were measured using the clinical IDEXX Vet Test chemistry analyser VT8008 (IDEXX Laboratories, West Brook, ME, USA).

### Cell culture

Activated human (LX2) and rat (HSC-T6) HSC cell lines were cultured in Dulbecco’s modified Eagle’s medium containing 10% foetal bovine serum (FBS), 100 μg/mL penicillin, and 0.25 μg/mL streptomycin (all from Invitrogen, Carlsbad, CA, USA). HepG2 cells were grown in minimum essential medium supplemented with 10% FBS, 100 μg/ml penicillin and 0.25 μg/ml streptomycin (all from Invitrogen). All cell lines were maintained at 37°C in a humidified incubator with 5% CO_2_. The antiviral drugs ETV (Sigma-Aldrich) and TDF (Santa Cruz Biotechnology, Dallas, Texas, USA) were dissolved in distilled water. At 24 h after seeding, the cells were treated with antiviral drugs or the autophagy inhibitor chloroquine (CQ) (20 μM).

### Statistical analysis

The data are expressed as the means ± standard deviation and were obtained from several separate experiments. Comparisons of means were made using Student’s t-test and were considered significant when *p < 0.05, **p < 0.01, and *** p < 0.001. Statistical analysis was performed using GraphPad Prism 7 software (GraphPad, San Diego, CA, USA). Methods regarding cell viability and apoptosis assays, quantitative real-time PCR, western blotting, the measurement of liver collagen, histological examinations and immunofluorescence staining are included as supplementary materials.

## Results

### TDF ameliorated liver fibrosis in a TAA-induced liver fibrosis mouse model

**Fig 1:**
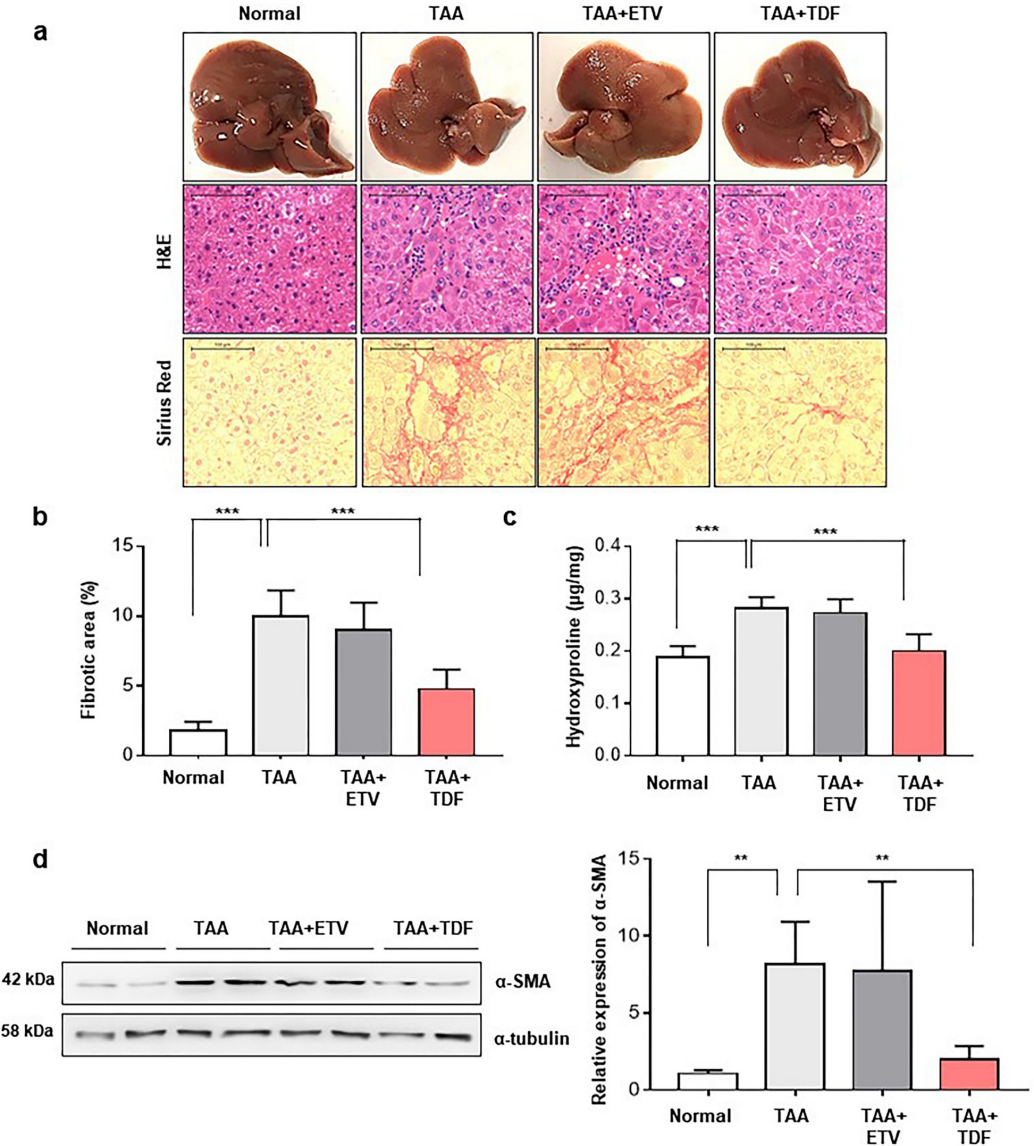
Antifibrotic effect of TDF in a TAA-induced liver fibrosis mouse model. (A) The effect of the antiviral drugs on TAA-induced fibrotic mouse livers was assessed. The liver tissues were stained with H&E and Sirius red. Original magnification: 100x. (B) Morphometric analysis was performed on Sirius Red-stained liver sections (n = 5 fields/liver) to measure collagen deposition. (C) The amount of liver hydroxyproline was evaluated (n = 5 section/liver) by hydroxyproline assay. (D) Expression of α-SMA was determined by western blotting. The relative expression was normalized to α-tubulin expression as a reference. The values are presented as the mean ± standard error of the mean. All data are representative of at least three independent experiments. TAA, thioacetamide; ETV, entecavir; TDF, tenofovir disoproxil fumarate; α-SMA, alpha smooth muscle actin *P<0.05, **P<0.01, ***P<0.001.

**Fig 2:**
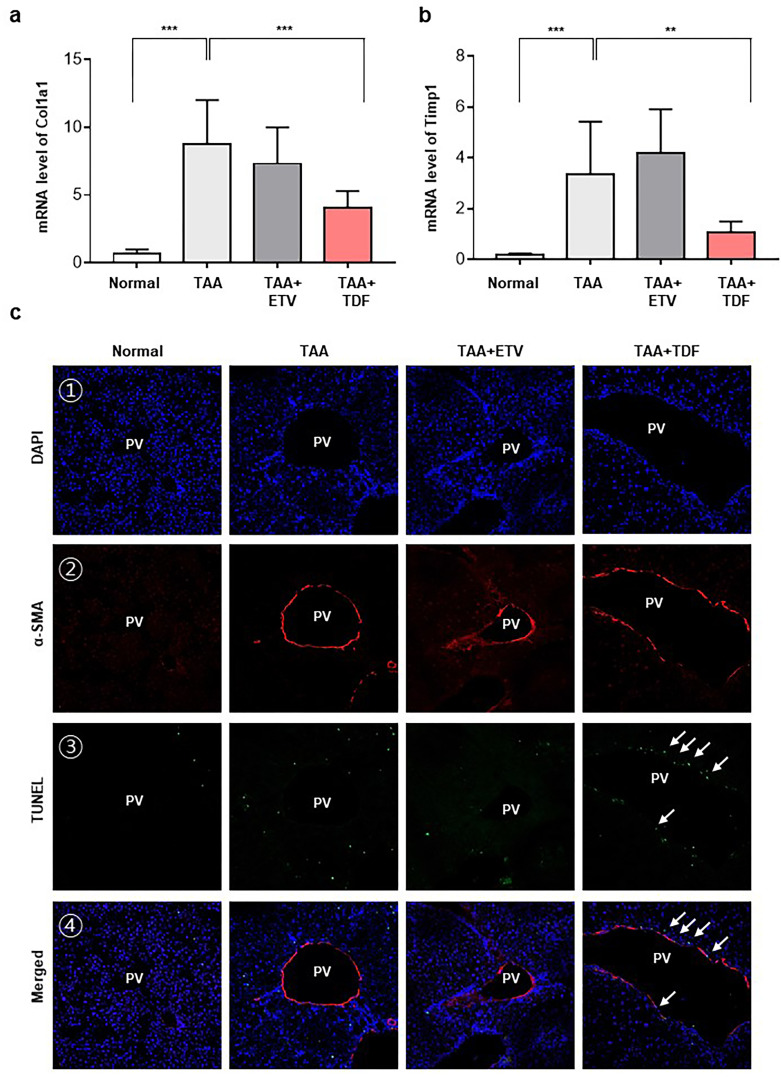
The effect of TDF on hepatic stellate cell apoptosis in the TAA-induced liver fibrosis mouse model. (A and B) The mRNA levels of Col1a1 and Timp1 were evaluated by qRT-PCR. (C) Confocal images of α-SMA and TUNEL staining in TAA-induced fibrotic mouse livers. (1) DAPI was used for nuclear staining, (2) α-SMA staining (red), and (3) TUNEL-positive nuclei (green). (4) Composite image of (1) to (3). Original magnification: 100x. All data are representative of at least three independent experiments. Col1α1, collagen1α1; TIMP, tissue inhibitor of metalloproteinases; TAA, thioacetamide; ETV, entecavir; TDF, tenofovir disoproxil fumarate; DAPI, 4’, 6-diamidino-2-phenylindole; α-SMA, alpha smooth muscle actin; TUNEL, terminal deoxynucleotidyl transferase dUTP nick-end labelling. *P<0.05, **P<0.01, ***P<0.001.

To verify the antifibrotic effect of TDF, we established a TAA-induced liver fibrosis mouse model. The mice received either TDF or ETV by oral gavage daily for 10 weeks. Haematoxylin and eosin (H&E) staining of livers from TDF-treated mice showed almost normal histology, whereas livers from the ETV-treated group showed sinusoidal congestion, hepatocellular necrosis, and lymphocyte infiltration. In addition, Sirius red staining showed that the collagen deposition area was significantly alleviated in the TDF group, whereas advanced liver fibrosis, the accumulation of a large amount of collagen, fibrosis expansion and marked bridging were observed in the ETV-treated group (Fig 1A and 1B). The hepatic hydroxyproline, a major constituent of collagen and a good marker of ECM accumulation [17], was also decreased significantly by TDF but not ETV (Fig 1C). Additionally, in the TDF group, the expression of the activated HSC marker alpha-smooth muscle actin (α-SMA) and the mRNA levels of collagen type Ⅰ alpha 1 (Col1α1) and TIMP-1 were dramatically decreased compared to those in the ETV group (Figs 1D and 2B). These results indicate that TDF could effectively ameliorate TAA-induced liver fibrosis *in vivo* by decreasing the number of activated hepatic stellate cells (HSCs).

### TDF induced HSC death in a TAA-induced liver fibrosis model

Reversion of fibrosis in the liver is closely associated with the clearance of HSC by apoptosis [18]. To verify the effect of TDF on the death of activated HSCs, we performed α-SMA staining and TUNEL assays in a TAA-induced liver fibrosis model. TUNEL-positive cells were observed in a randomly scattered pattern in the TAA and ETV groups. In contrast, TUNEL-positive cells in the TDF group were concentrated around the portal vein tract in the liver. Considering that liver fibrosis is initiated at the periportal area, the TUNEL-positive cells in the TDF group were specifically localized at the periportal fibrotic area (Fig 2C). We performed α-SMA staining to confirm whether the TUNEL-positive cells overlapped with the α-SMA-positive cells, which represent activated HSCs in fibrotic areas. As a result, α-SMA-positive cells were observed in the periportal areas of all groups, but only in the TDF group did the TUNEL-positive cells overlap with the α-SMA stain. These results indicate that the administration of TDF specifically induces cell death in HSCs.

To assess the cytotoxicity of the doses of TDF used in our study, serum ALT and creatinine were measured to investigate whether TDF could induce significant apoptosis in not only HSCs but hepatocytes and renal cells. The serum ALT levels that were elevated by TAA injection were significantly reduced by TDF treatment. Additionally, the serum creatinine levels remained within the normal range after TDF treatment. These results indicate that the administration of TDF at the dose used in our study did not have any adverse impact on liver or kidney function (S1 Fig). Collectively, in our liver fibrosis mouse model, TDF selectively induced cell death in activated HSCs without notable adverse systemic effects.

### TDF inhibited the proliferation and decreased the viability of HSCs

**Fig 3:**
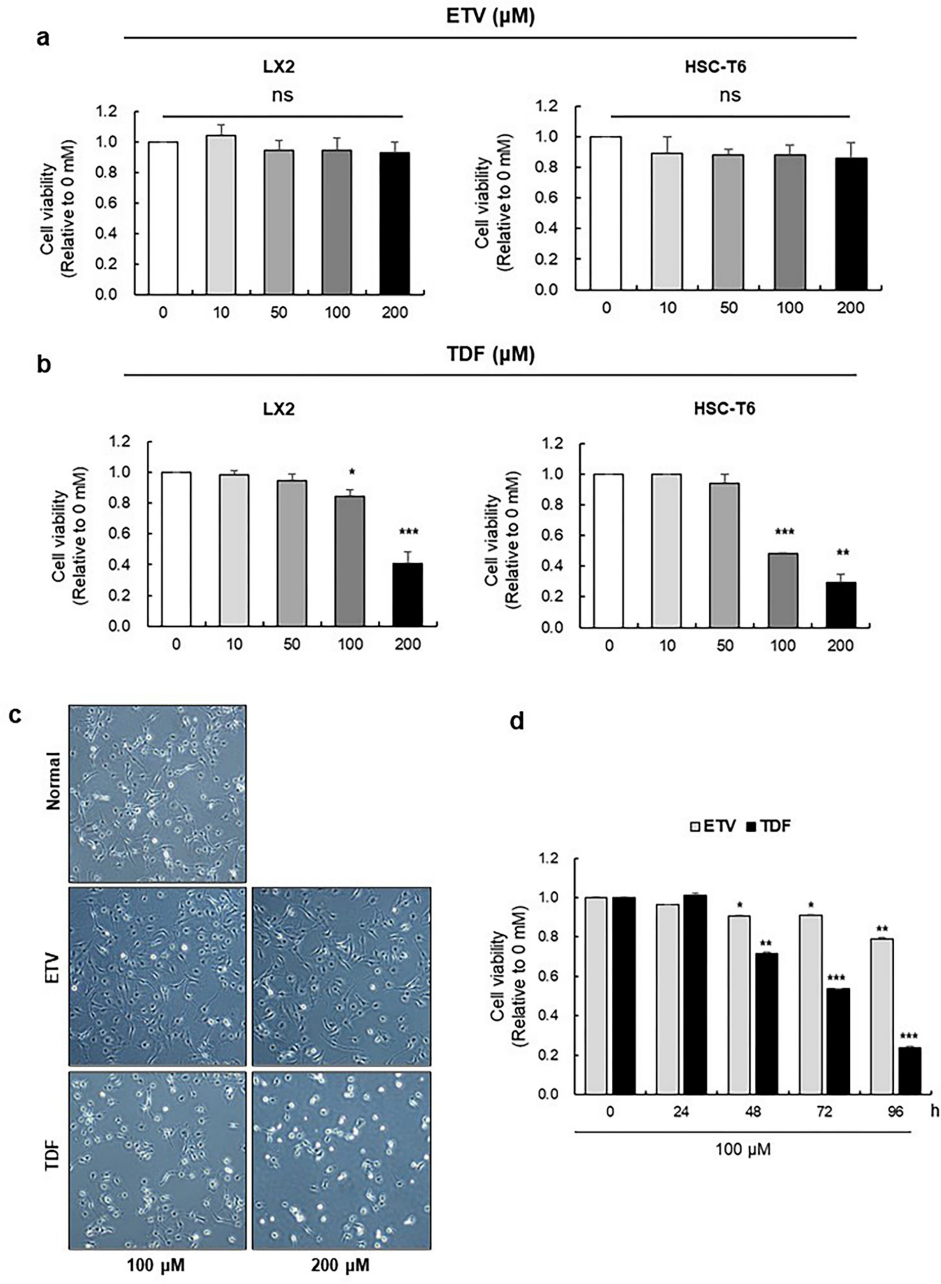
Treatment with TDF decreased cell viability and induced morphological changes in hepatic stellate cells. (A and B) Human and rat hepatic stellate cell lines (LX2 and HSC-T6) were treated with 0, 10, 50, 100 and 200 μM ETV or TDF for 24 h. (C) Using phase-contrast imaging, morphological changes were assessed in LX2 cells after treatment with 100 and 200 μM ETV or TDF for 24 h (original magnification: 100x). (D) LX2 cells were treated with 100 μM ETV or TDF for 96 h. All data are representative of at least three independent experiments. NS, not significant; ETV, entecavir; TDF, tenofovir disoproxil fumarate. *P<0.05, **P<0.01, ***P<0.001.

To investigate the direct impact of TDF on activated HSCs in liver fibrosis, we treated TDF on LX2 and HSC-T6 hepatic stellate cell lines. TDF treatment induced significant reductions in the viabilities of both cell lines at a dose of 100 μM and a greater reduction at a dose of 200 μM after 24 h, but no significant changes were observed in ETV-treated cells (Fig 3A and 3B). Additionally, phase contrast microscopy showed a decrease in cell number and an increase in cell shrinkage and transformation to round-shaped cells, which are typical features of apoptosis, in TDF-treated LX2 cells but not ETV-treated cells (Fig 3C) [19]. As 200 μM TDF was associated with an abrupt reduction in cell viability, we performed two additional experiments to determine whether HSC death was caused by the cytotoxic effects of high-dose TDF. First, we treated cells with 100 μM TDF or ETV for 96 h. Even after treatment with 100 μM, the viability of LX2 cells decreased significantly in a time-dependent manner, with a 76% decrease at 96 h after TDF administration. In contrast, the survival rate of LX2 cells decreased modestly by 21% in the ETV group (Fig 3D). Then, we treated HepG2 cells with TDF and compared the viability with that of HSCs. The viability of HepG2 cells remained higher than 85% after TDF treatment, even at a dose of 200 μM, compared to the significant decrease in LX2 cell viability (S2 Fig). Then, we isolated HSCs and hepatocytes from TAA-induced fibrotic mice liver to confirm the cell viabilities following treatment of TDF and ETV. The results showed that the cell viability of HSCs were down-regulated in only TDF-treated HSCs whereas in ETV-treated HSC and even in TDF-treated hepatocytes were not changed (S3 Fig). These results suggest that TDF specifically inhibits the proliferation and decreases the viability of HSCs but not hepatocytes.

### TDF induced cell death in HSCs through the apoptosis pathway

**Fig 4:**
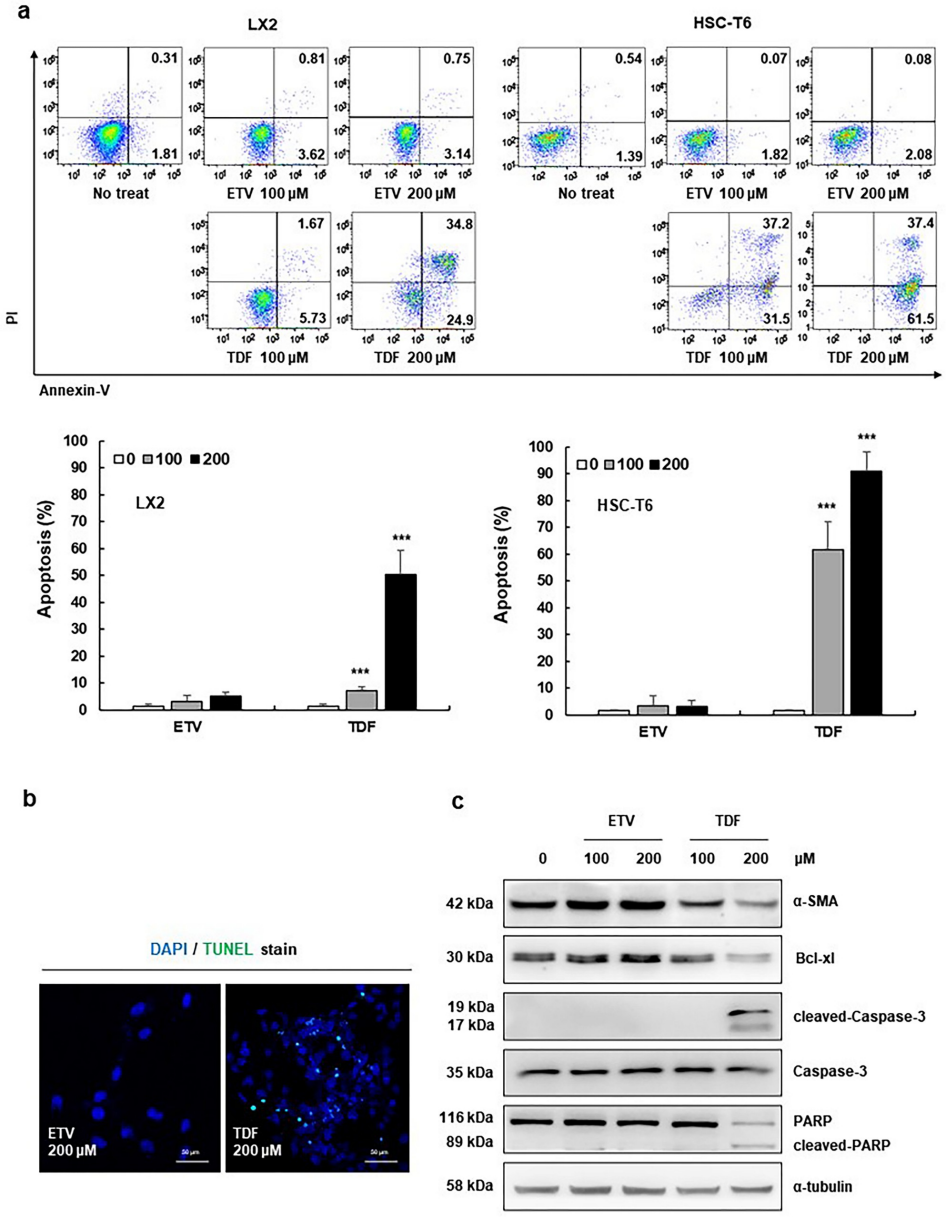
TDF induced hepatic stellate cell death through the apoptosis pathway. (A) LX2 and HSC-T6 cells were treated with 100 or 200 μM ETV or TDF for 24 h. Apoptotic cells were assessed by flow cytometry, and the percentage of apoptotic cells was calculated. (B) Measurement of apoptotic LX2 cells using TUNEL staining after treatment with ETV or TDF. Green fluorescence indicates TUNEL-positive cells. Cells were co-stained with DAPI to identify the cell nuclei. (original magnification: 200x). (C) The expression of α-SMA, cleaved caspase-3, Caspase-3, PARP, cleaved-PARP, and Bcl-xl in LX2 cells was determined by western blotting. The relative expression was normalized to α-tubulin expression as a reference. All data are representative of at least three independent experiments. ETV, entecavir; TDF, tenofovir disoproxil fumarate; TUNEL, terminal deoxynucleotidyl transferase dUTP nick-end labelling; α-SMA, alpha smooth muscle actin; PARP, poly (ADP-ribose) polymerase; Bcl-xl, B-cell lymphoma-extra large. ***P<0.001.

Because TDF-treated HSCs exhibited typical features of cells undergoing apoptosis, we conducted an Annexin V-propidium iodide (Annexin V-PI) assay to analyse the apoptotic cell rates after treatment with TDF and ETV for 24 h. Treatment with 100 μM and 200 μM TDF significantly increased the rate of apoptosis in both cell lines. In LX2 cells, the percentage of apoptotic cells was significantly increased in the 200 μM TDF group (57.7%) compared to that of the ETV group (3.89%). Additionally, in HSC-T6 cells, the percentage of apoptotic cells was 98.9% after TDF treatment and 2.16% after ETV treatment. In particular, early apoptotic LX2 cells (Annexin V-positive and PI-negative) were predominant after TDF treatment, indicating that HSC death was induced by apoptosis rather than unprogrammed cell death (Fig 4A). In addition, confocal microscopy images showed an increase in TUNEL-positive cells in the TDF group but not the ETV group (Fig 4B). To determine the apoptosis signalling pathways stimulated by TDF treatment, the apoptosis-associated proteins PARP, Caspase-3 and Bcl-xl were analysed in LX2 and HSC-T6 cells after treatment with 100 and 200 μM TDF or ETV. Only in the TDF group was an increase in PARP and Caspase-3 fragments and a decrease in the anti-apoptotic protein Bcl-xl detected (Fig 4C and S4 Fig). These findings indicate that TDF induces apoptosis in HSCs through the regulation of apoptosis-associated proteins.

### TDF increased autophagy in HSCs

**Fig 5:**
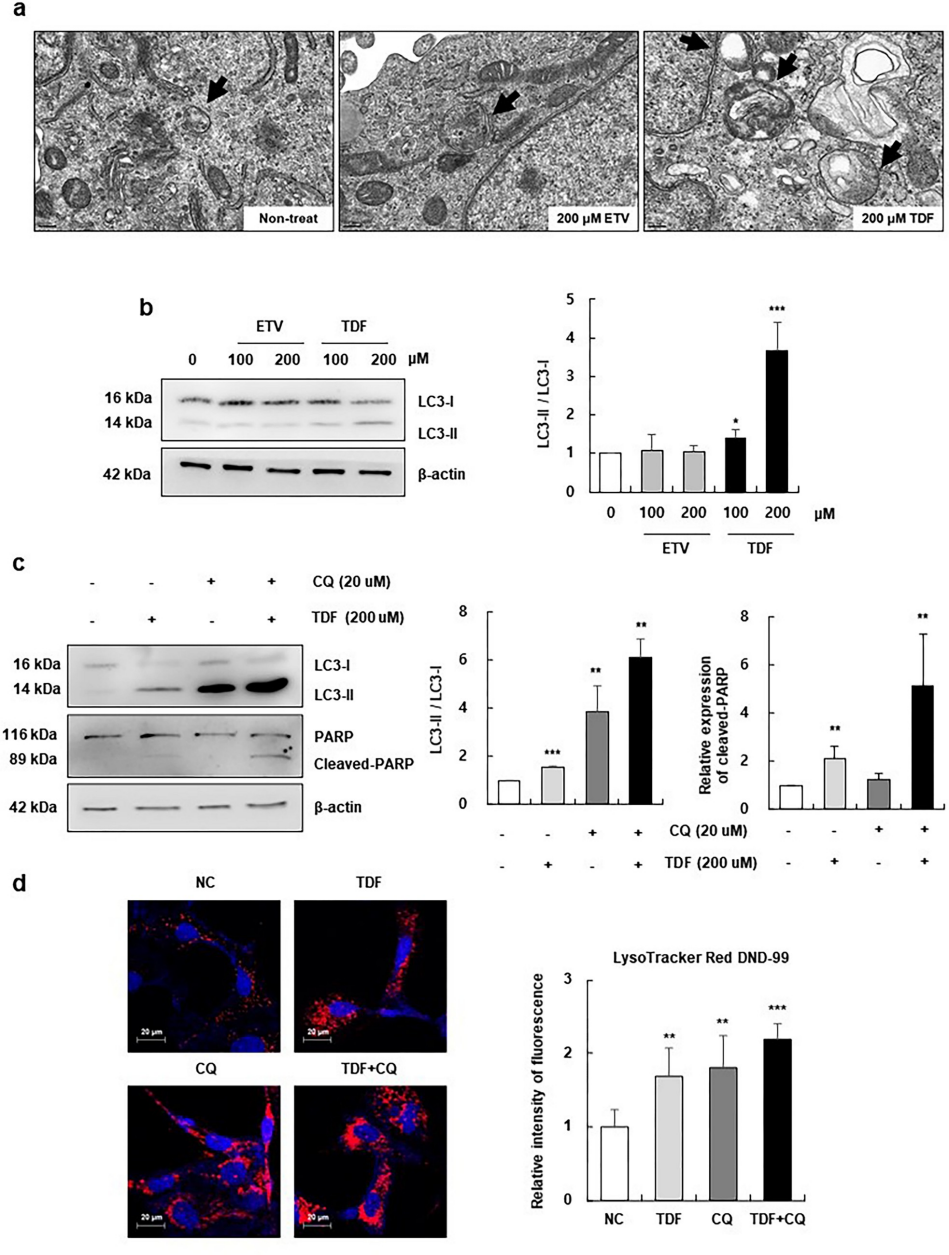
TDF increased autophagy in hepatic stellate cells. (A) Transmission electron microscopy revealed the numbers of autophagosomes in LX2 cells incubated with ETV or TDF for 24 h. Arrows indicate autophagosome structures. (B) The expression of LC3-I and LC3-II in LX2 cells was determined by western blotting. (C) The expression of LC3-I, LC3-II and PARP in CQ- and TDF-treated LX2 cells was determined by western blotting. The relative expression of LC3-II was normalized to LC3-I expression, and the relative expression of cleaved PARP was normalized to β-actin expression as a reference. (D) TDF- and CQ-treated LX2 cells were stained with LysoTracker, and fluorescence was observed under a confocal microscope; fluorescence intensity was quantified using a Zen 2.6 blue edition. All data are representative of at least three independent experiments. ETV, entecavir; TDF, tenofovir disoproxil fumarate; CQ, chloroquine; PARP, poly (ADP-ribose) polymerase; NC, negative control. *P< 0.05, **P< 0.01, ***P< 0.001.

Transmission electron microscopy examination showed a substantial increase in autophagosomes after TDF treatment but not ETV treatment (Fig 5A). Western blot analysis showed a significantly higher LC3-II/LC3-I ratio in the TDF group than in the ETV group, indicating an increase in autophagy (Fig 5B and S5 Fig). Next, to determine the impact of autophagy on HSC apoptosis, we compared the effect of TDF to the classic autophagy inhibitor CQ. After treating cells with TDF and CQ simultaneously, the levels of LC3-II and cleaved PARP increased compared to those of cells treated with TDF or CQ alone. These results suggest that TDF treatment induced an increase in both autophagy and apoptosis (Fig 5C). Then, we examined markers of the lysosomal membrane using LysoTracker Red DND-99 (a commercially available lysosome‐targeting dye), as the fusion of lysosomes and autophagosomes is the penultimate step of the autophagic pathway. As a result, the fluorescence intensity of LysoTracker increased significantly after TDF treatment similar to that of CQ treatment, and higher fluorescence intensity was observed after TDF and CQ cotreatment than that of TDF treatment alone (Fig 5D). These results collectively indicate that TDF induces both autophagy and apoptosis in HSCs and that both processes regulate programmed cell death in LX2 cells through crosstalk.

### TDF-induced apoptosis and autophagy were dependent upon inhibition of the PI3K/Akt/mTOR pathway

**Fig 6:**
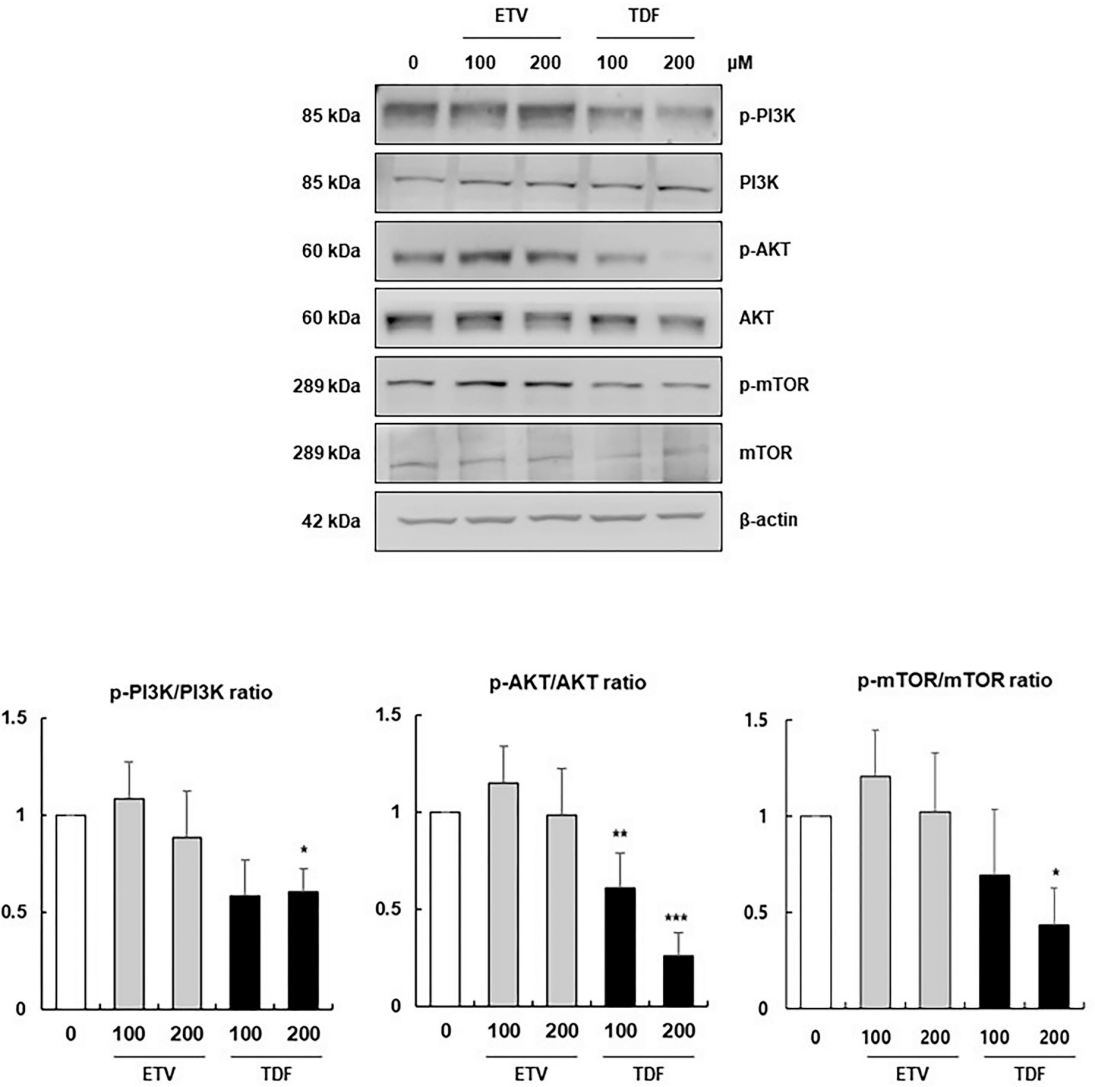
The effect of TDF on the PI3K/Akt/mTOR signalling pathway. The expression of PI3K, Akt and mTOR in LX2 cells was analysed by western blotting. The relative expression of phosphorylated proteins was normalized to the total protein expression. All data are representative of at least three independent experiments. ETV, entecavir; TDF, tenofovir disoproxil fumarate; PI3K, phosphoinositide 3-kinases; Akt, protein kinase B; mTOR, mammalian target of rapamycin. *P<0.05, **P<0.01, ***P<0.001.

To identify the inducer of TDF-mediated autophagy and apoptosis, we further investigated the effect of TDF on the PDGF and TGF-β signalling pathways, such as PI3K/Akt/mTOR, Smad and mitogen-activated protein kinase (MAPK), which have been reported to regulate cell proliferation and fibrogenesis in HSCs. The results showed that the phosphorylation of PI3K/Akt/mTOR was consistently and significantly decreased after treatment with TDF, whereas treatment with ETV did not have a significant effect (Fig 6 and S6 Fig). However, the expression of the TGF-β signalling-related proteins Smad2/3, extracellular signal-regulated kinases (ERK), c-Jun N-terminal kinases (JNK) and p38 MAPK was not affected by TDF or ETV treatment (S7 Fig). These results demonstrate that TDF exerts its antifibrotic effect by inducing HSC apoptosis by inhibiting the phosphorylation of PI3K/Akt/mTOR signalling pathway.

## Discussion

In the present study, we attempted to clarify the mechanisms by which the antiviral agent TDF exerts antifibrotic effects both *in vitro* and in an *in vivo* liver fibrosis model. After TDF treatment, the degree of fibrosis and the number of activated HSCs were significantly reduced in the liver tissue of the hepatic fibrosis mouse model. Additionally, apoptotic cells were localized in the fibrotic periportal area, which stained positive for α-SMA in the merged images, suggesting that TDF induces apoptosis in activated HSCs, resulting in a specific antifibrotic effect. An *in vitro* study to elucidate the precise mechanism of these findings revealed that 1) TDF inhibited the proliferation and decreased the viability of HSCs; 2) TDF increased autophagy in HSCs; and 3) TDF may induce HSC apoptosis by inhibiting the phosphorylation of PI3K/Akt/mTOR signalling pathway components. These results suggested that TDF induced an antifibrotic effect in activated HSCs by coordinating the regulation and crosstalk between apoptosis and autophagy.

Regression of hepatic fibrosis has been reported clinically in various liver diseases after treatment or the removal of the causative agents [20–22]. A definite decrease in hepatic inflammation and the potential regenerative capacity of the damaged liver may allow for normal restoration of the liver. However, there is a limit to the complete regression of hepatic fibrosis only by these mechanisms. Therefore, reinforcement of other antifibrotic mechanisms that target activated hepatic stellate cells is essential. Previous studies have shown that anticancer agents such as sorafenib and nilotinib exert antifibrotic effects through activated HSC apoptosis [9, 23]. Although such findings were promising, the high toxicity profiles of anticancer agents prevented these drugs from being used as antifibrotic agents. Our data may have a significant impact, as we investigated and compared the direct antifibrotic effects of the two most widely used antiviral agents for the treatment of CHB. TDF, which showed significant antifibrotic activity through inducing HSC apoptosis in this study, exhibited relatively minor toxicity even after long-term use [24].

Furthermore, TDF induced HSC apoptosis by inhibiting the phosphorylation of PI3K/Akt/mTOR signalling pathway factors, which were shown to be associated with the activation of HSCs and fibrogenesis in previous studies. Marra et al. showed that PI3K activation was necessary for both mitogenesis and chemotaxis in PDGF-induced HSCs [25]. Following the activation of PI3K, Akt kinase activity and P70^S6K^/mTOR signalling are induced, which have been reported to be associated with HSC proliferation and α1 collagen transcription and translation [26–28]. Additionally, Peso et al. showed that activated Akt inhibits the phosphorylation of Bcl-2-associated agonist of cell death (BAD) [29]. BAD is a proapoptotic member of the Bcl-2 family that, upon phosphorylation, loses its ability to inhibit the antiapoptotic protein Bcl-xl and therefore reduces cell death. Correspondingly, pharmacological PI3K inhibitors such as HS-173 and LY294002 or an adenovirus expressing a dominant negative form of PI3K (Ad-SMAdnPI3K) were found to attenuate HSC activation and block the progression of liver fibrosis [30, 31]. Our study showed that TDF, which was originally an antiviral drug, was associated with the inhibition of p-PI3K, p-Akt and p-mTOR expression, which led to the downregulation of Bcl-xl and consequently HSC apoptosis to ameliorate liver fibrosis both *in vitro* and *in vivo*. Recently, Murata et al. reported that TDF impaired Akt phosphorylation to enhance anti-HBV immunity, which supports our data that TDF is associated with the inhibition of Akt [32]. However, our study was different in that we demonstrated novel findings associated with the antifibrotic effects of TDF and its underlying mechanisms.

Then, we investigated the effect of TDF on autophagy to thoroughly evaluate the role of TDF in the mechanisms of HSC death, as apoptosis and autophagy are the two major modes of cell death that occur to maintain homeostasis or in pathologic conditions [33, 34]. The functional relationship between autophagy and apoptosis is known to be complex, as autophagy can either promote or inhibit apoptosis depending on the type or duration of the stimulus and cell context even within the same conditions [23, 35]. Previously, the PI3K/Akt/mTOR signalling pathway was reported to have an inhibitory effect on autophagy [36, 37]. In the same context, pharmacological inhibitors of the PI3K/Akt/mTOR pathway, LY294002 and triptolide, were found to alleviate hepatic and renal fibrosis, respectively, by enhancing autophagy [35, 38]. Accordingly, our data showed an increase in the autophagosome marker LC3-II following TDF treatment (Fig 3B). Similar to TDF, nilotinib has been reported to induce both apoptosis and autophagic cell death in activated HSCs simultaneously [23]. Also, the addition of CQ, a classic autophagy inhibitor, resulted in a further increase in cleaved PARP, a marker of apoptosis (Fig 3C). Therefore, it may be possible that the TDF-induced increase in autophagy may also act as a regulator of apoptosis in LX2 cells through crosstalk.

Currently, there is ongoing controversy regarding the risk of hepatocellular carcinoma (HCC) and mortality in response to TDF and ETV, since Choi et al. first suggested that TDF was associated with a lower risk of HCC than ETV [39]. In contrast, we demonstrated that there was no difference between the two drugs regarding the risk of HCC and mortality [40]. Papatheodoridis et al. also suggested that there was no difference between the two drugs but interestingly demonstrated that elastographic reversion at year 5 was more frequent in TDF- than ETV-treated patients with pretreatment cirrhosis. Therefore, it may be possible in the long term that the direct antifibrotic effect of TDF may promote additional beneficial effects [15, 41, 42]. The crucial remaining issue is discovering the effective and safe antifibrotic dose of TDF in humans. In our experiment, we administered 100 mg/kg/day TDF by oral gavage to mice, which is approximately 1–1.5-fold higher than the standard adult dose. Similarly, 75 mg/kg/day TDF by subcutaneous injection in mice has been reported to attenuate liver fibrosis [43]. Furthermore, the safety of long-term administration of the antifibrotic dose of TDF is equally important, as TDF has been associated with renal toxicity and decreased bone mineral density in some patients [44]. However, our data showed no apparent renal toxicity in response to 100 mg/kg/day TDF in mice.

There were a few limitations in this study. First, the precise chemical structure of TDF that is associated with HSC apoptosis and the corresponding binding site on HSCs are still unknown. However, it has been recently suggested that TDF blocks Pannexin-1, which results in decreased adenosine levels and hence diminishes adenosine a2 receptor (A2AR)-mediated skin and liver fibrosis [43]. A2AR antagonism has also been reported to decrease fibrosis in ethanol-exacerbated liver fibrosis [45]. In addition, an increase in adenosine levels has been reported to interact with A2AR to enhance PI3K/Akt/mTOR pathway signalling [46]. Therefore, it may be possible that TDF downregulates adenosine and A2AR levels, which results in the inhibition of PI3K/Akt/mTOR phosphorylation and ultimately leads to HSC apoptosis and the amelioration of liver fibrosis. Second, there is no clinical evidence of TDF as an antifibrotic drug in humans with no head-to-head randomized controlled trial comparing the antifibrotic effects of ETV and TDF. Therefore, the optimal dose, treatment duration and safety of antifibrotic TDF therapy have yet to be determined. Interestingly, in terms of safety issues, the decrease in bone mineral density induced by TDF has also been reported to be associated with a decrease in adenosine levels and the downregulation of A2AR [47]. Therefore, it will be very important to assess the benefits and possible disadvantages of TDF treatment with respect to antifibrotic and possible adverse effects related to A2AR inhibition. Tenofovir alafenamide, which has shown additional safety and equal effectiveness compared to TDF, may be an option for further studies. Third, the apoptosis and autophagy pathways of HSCs following TDF treatment were only evaluated in vitro. The strengths of this study were that it was the first study to compare the antifibrotic effects of TDF and ETV, which is important regarding the current controversies, and we comprehensively performed detailed analyses on the underlying mechanisms associated with TDF and programmed cell death in HSCs, including apoptosis, autophagy, and upstream pathways.

In conclusion, this study demonstrated that TDF directly ameliorates liver fibrosis through the downregulation of the PI3K/Akt/mTOR signalling pathway, which results in the apoptosis of activated HSCs. The antifibrotic effects of TDF indicate that it may be a therapeutic agent for the treatment of liver fibrosis. Further human studies are necessary to discover the safe and effective antifibrotic dose of TDF in CHB patients.

## Supporting information

S1 Fig Serum ALT and creatinine levels following TDF treatment in a TAA-induced liver fibrosis mouse model. (A) Serum ALT levels in the control, TAA-induced liver injury, TAA+TDF treatment, and TAA+ETV treatment groups. (B) Serum creatinine levels in the control, TAA-induced liver injury, TAA+TDF treatment, and TAA+ETV treatment groups. ALT, alanine aminotransferase; TAA, thioacetamide; ETV, entecavir; TDF, tenofovir disoproxil fumarate. *P< 0.05, **P< 0.01, ***P< 0.001.
(DOCX)

S2 Fig TDF specifically inhibits proliferation and decreases the viability of hepatic stellate cells but not hepatocytes. HepG2 and LX2 cells were treated with various concentrations of TDF for 24 h and analyzed with the MTT assay. All data are representative of at least three independent experiments. *P< 0.05, **P< 0.01, ***P< 0.001.
(DOCX)

S3 Fig Treatment with TDF decreased cell viability and induced morphological changes in isolated hepatic stellate cells from fibrotic mouse liver. (A) Isolated hepatocytes and hepatic stellate cells were treated with 100 uM ETV or TDF for 24 h. (B) Using phase-contrast imaging, morphological changes were assessed in isolated hepatocytes and hepatic stellate cells after treatment with 100 μM ETV or TDF for 24 h (original magnification: 100x).
(DOCX)

S4 Fig TDF induced hepatic stellate cell death through the apoptosis pathway. The expression of α-SMA, Bcl-xl, cleaved caspase-3, caspase-3, PARP and cleaved-PARP in HSC-T6 cells was determined by western blotting. The relative expression was normalized to α-tubulin expression as a reference. ETV, entecavir; TDF, tenofovir disoproxil fumarate; α-SMA, alpha smooth muscle actin; Bcl-xl, B-cell lymphoma-extra large; PARP, poly (ADP-ribose) polymerase.
(DOCX)

S5 Fig TDF increased autophagy in hepatic stellate cells. The expression of LC3-I and LC3-II in HSC-T6 cells was determined by western blotting. The relative expression of LC3-II was normalized to LC3-I expression. ETV, entecavir; TDF, tenofovir disoproxil fumarate.
(DOCX)

S6 Fig The effect of TDF on the PI3K/Akt/mTOR signalling pathway. The expression of PI3K, Akt and mTOR in HSC-T6 cells was analysed by western blotting. ETV, entecavir; TDF, tenofovir disoproxil fumarate; PI3K, phosphoinositide 3-kinases; Akt, protein kinase B; mTOR, mammalian target of rapamycin.
(DOCX)

S7 Fig The effect of TDF on TGF-β signalling pathway components in LX2 cells. (A and B) Smad or non-Smad signalling pathway proteins in LX2 cells were measured by western blotting. All data are representative of at least three independent experiments. ERK, extracellular signal-regulated kinase; ETV, entecavir; JNK, c-Jun N-terminal kinase; TDF, tenofovir disoproxil fumarate; TGF-β, transforming growth factor beta.
(DOCX)

S8 Fig Original uncropped and unadjusted images. (DOCX)

S1 Table Primary antibodies used for western blot. (DOCX)

S1 File Supplementary methods. (DOCX)

S2 File Minimal data set underlying the results described in the manuscript. (DOCX)
