# Supplementary material for: Tenofovir disoproxil fumarate directly ameliorates liver fibrosis by inducing hepatic stellate cell apoptosis via downregulation of PI3K/Akt/mTOR signaling pathway
Source: PLoS One. 2021 Dec 8;16(12):e0261067. doi: 10.1371/journal.pone.0261067 (PMC8654182; doi:10.1371/journal.pone.0261067)
Supplement: S1 Fig — (A) Serum ALT levels in the control, TAA-induced liver injury, TAA+TDF treatment, and TAA+ETV treatment groups. (B) Serum creatinine levels in the control, TAA-induced liver injury, TAA+TDF treatment, and TAA+ETV treatment groups. ALT, alanine aminotransferase; TAA, thioacetamide; ETV, entecavir; TDF, tenofovir disoproxil fumarate. *P< 0.05, **P< 0.01, ***P< 0.001. (DOCX) [file pone.0261067.s001.docx]

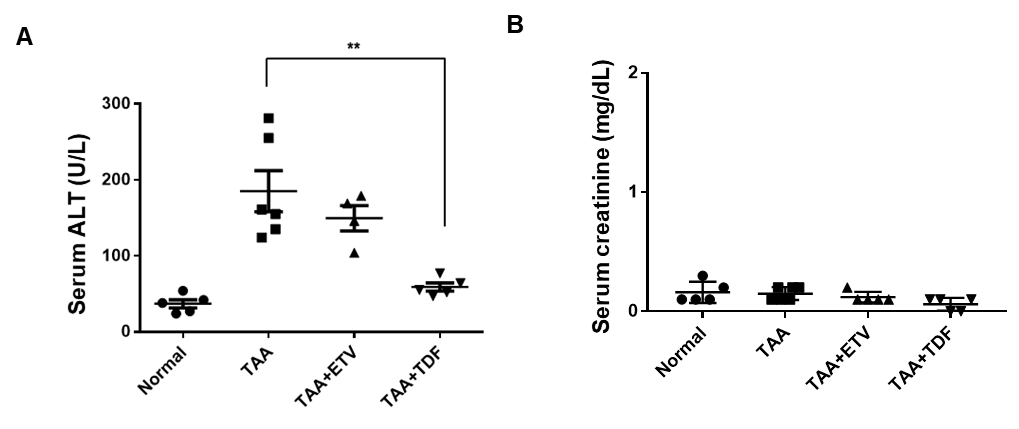


**Supplementary Fig 1. Serum ALT and creatinine levels following TDF treatment in a TAA-induced liver fibrosis mouse model.**

(A) Serum ALT levels in the control, TAA-induced liver injury, TAA+TDF treatment, and TAA+ETV treatment groups. (B) Serum creatinine levels in the control, TAA-induced liver injury, TAA+TDF treatment, and TAA+ETV treatment groups. ALT, alanine aminotransferase; TAA, thioacetamide; ETV, entecavir; TDF, tenofovir disoproxil fumarate. *P< 0.05, **P< 0.01, ***P< 0.001.
