# Supplementary material for: Tenofovir disoproxil fumarate directly ameliorates liver fibrosis by inducing hepatic stellate cell apoptosis via downregulation of PI3K/Akt/mTOR signaling pathway
Source: PLoS One. 2021 Dec 8;16(12):e0261067. doi: 10.1371/journal.pone.0261067 (PMC8654182; doi:10.1371/journal.pone.0261067)
Supplement: S2 Fig — HepG2 and LX2 cells were treated with various concentrations of TDF for 24 h and analyzed with the MTT assay. All data are representative of at least three independent experiments. *P< 0.05, **P< 0.01, ***P< 0.001. (DOCX) [file pone.0261067.s002.docx]

**
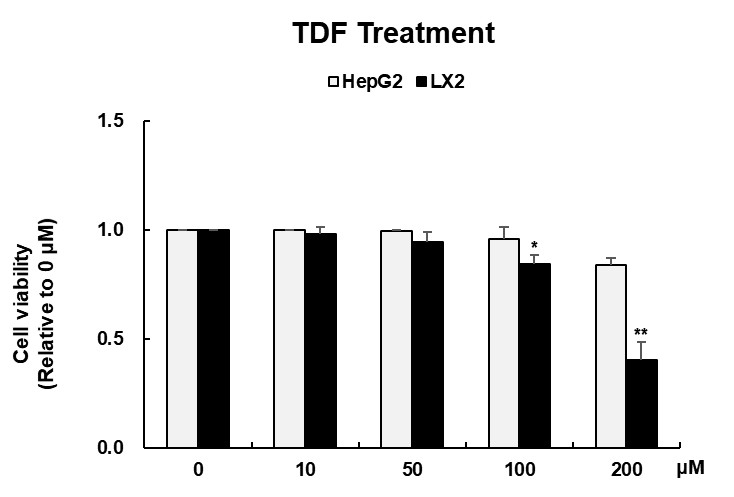
**

**Supplementary Fig 2. TDF specifically inhibits proliferation and decreases the viability of hepatic stellate cells but not hepatocytes**

HepG2 and LX2 cells were treated with various concentrations of TDF for 24 h and analyzed with the MTT assay. All data are representative of at least three independent experiments.

*P< 0.05, **P< 0.01, ***P< 0.001.
