# Supplementary material for: Tenofovir disoproxil fumarate directly ameliorates liver fibrosis by inducing hepatic stellate cell apoptosis via downregulation of PI3K/Akt/mTOR signaling pathway
Source: PLoS One. 2021 Dec 8;16(12):e0261067. doi: 10.1371/journal.pone.0261067 (PMC8654182; doi:10.1371/journal.pone.0261067)
Supplement: S3 Fig — (A) Isolated hepatocytes and hepatic stellate cells were treated with 100 uM ETV or TDF for 24 h. (B) Using phase-contrast imaging, morphological changes were assessed in isolated hepatocytes and hepatic stellate cells after treatment with 100 μM ETV or TDF for 24 h (original magnification: 100x). (DOCX) [file pone.0261067.s003.docx]

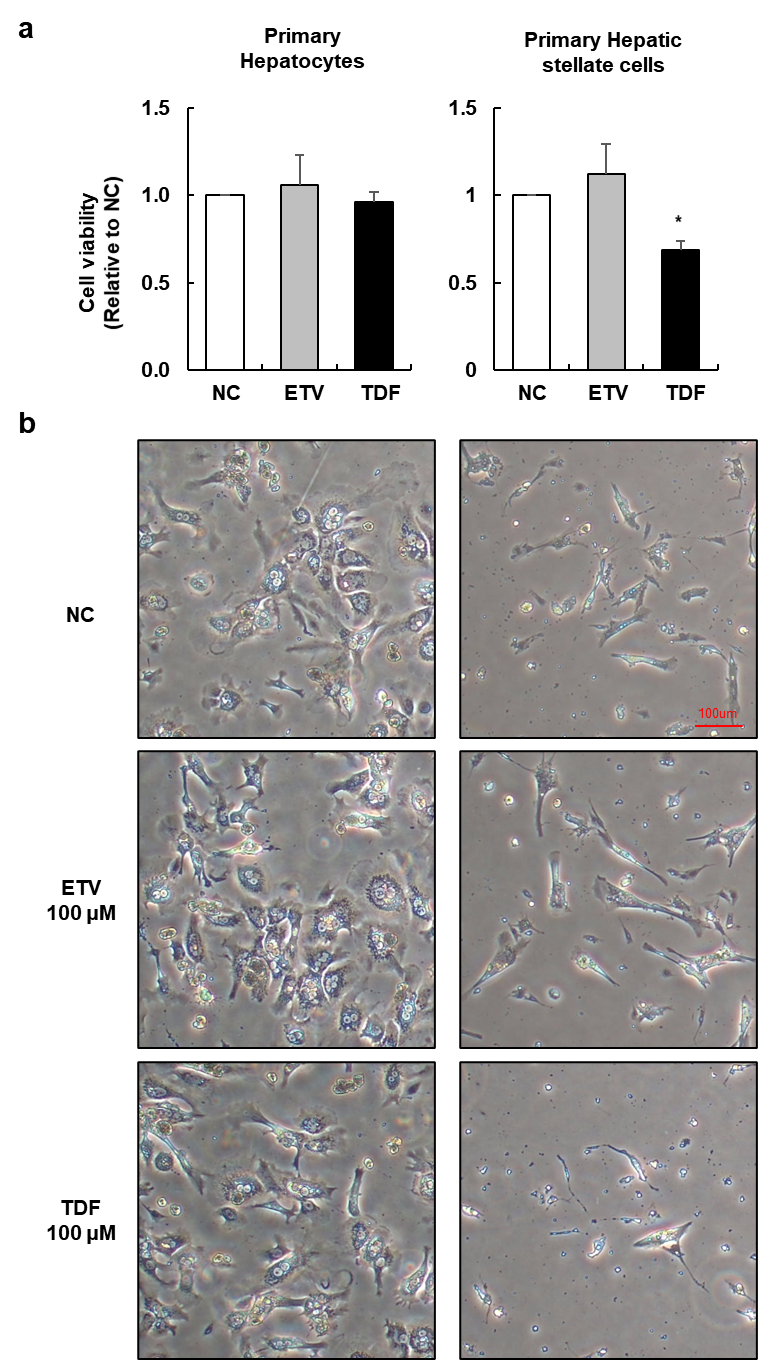


**Supplementary Fig 3. Treatment with TDF decreased cell viability and induced morphological changes in isolated hepatic stellate cells from fibrotic mouse liver.**

(A) Isolated hepatocytes and hepatic stellate cells were treated with 100 uM ETV or TDF for 24 h. (B) Using phase-contrast imaging, morphological changes were assessed in isolated hepatocytes and hepatic stellate cells after treatment with 100 μM ETV or TDF for 24 h (original magnification: 100x).
