# Supplementary material for: Tenofovir disoproxil fumarate directly ameliorates liver fibrosis by inducing hepatic stellate cell apoptosis via downregulation of PI3K/Akt/mTOR signaling pathway
Source: PLoS One. 2021 Dec 8;16(12):e0261067. doi: 10.1371/journal.pone.0261067 (PMC8654182; doi:10.1371/journal.pone.0261067)
Supplement: S4 Fig — The expression of α-SMA, Bcl-xl, cleaved caspase-3, caspase-3, PARP and cleaved-PARP in HSC-T6 cells was determined by western blotting. The relative expression was normalized to α-tubulin expression as a reference. ETV, entecavir; TDF, tenofovir disoproxil fumarate; α-SMA, alpha smooth muscle actin; Bcl-xl, B-cell lymphoma-extra large; PARP, poly (ADP-ribose) polymerase. (DOCX) [file pone.0261067.s004.docx]

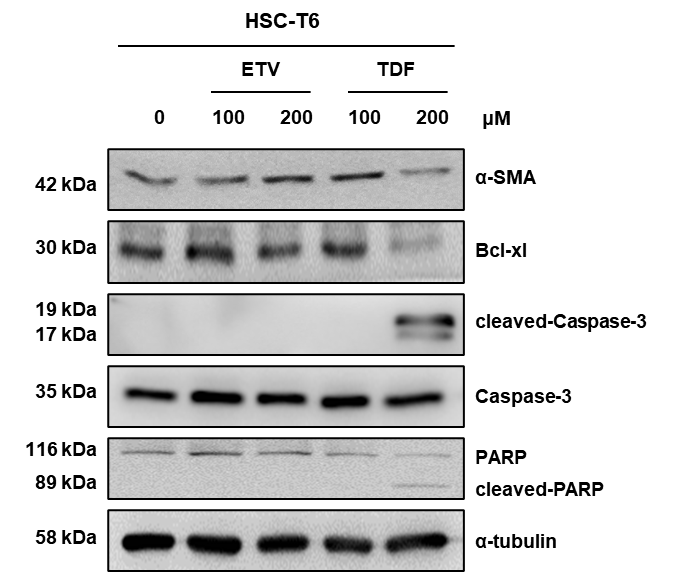


**Supplementary Fig 4. TDF induced hepatic stellate cell death through the apoptosis pathway.**

The expression of α-SMA, Bcl-xl, cleaved caspase-3, caspase-3, PARP and cleaved-PARP in HSC-T6 cells was determined by western blotting. The relative expression was normalized to α-tubulin expression as a reference. ETV, entecavir; TDF, tenofovir disoproxil fumarate; α-SMA, alpha smooth muscle actin; Bcl-xl, B-cell lymphoma-extra large; PARP, poly (ADP-ribose) polymerase.
