# Supplementary material for: Tenofovir disoproxil fumarate directly ameliorates liver fibrosis by inducing hepatic stellate cell apoptosis via downregulation of PI3K/Akt/mTOR signaling pathway
Source: PLoS One. 2021 Dec 8;16(12):e0261067. doi: 10.1371/journal.pone.0261067 (PMC8654182; doi:10.1371/journal.pone.0261067)
Supplement: S5 Fig — The expression of LC3-I and LC3-II in HSC-T6 cells was determined by western blotting. The relative expression of LC3-II was normalized to LC3-I expression. ETV, entecavir; TDF, tenofovir disoproxil fumarate. (DOCX) [file pone.0261067.s005.docx]

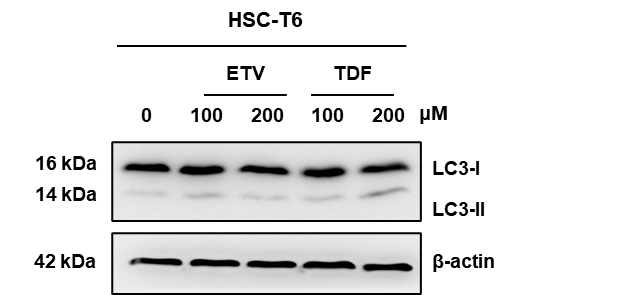


**Supplementary Fig 5. TDF increased autophagy in hepatic stellate cells.**

The expression of LC3-I and LC3-II in HSC-T6 cells was determined by western blotting. The relative expression of LC3-II was normalized to LC3-I expression. ETV, entecavir; TDF, tenofovir disoproxil fumarate.
