# Supplementary material for: Tenofovir disoproxil fumarate directly ameliorates liver fibrosis by inducing hepatic stellate cell apoptosis via downregulation of PI3K/Akt/mTOR signaling pathway
Source: PLoS One. 2021 Dec 8;16(12):e0261067. doi: 10.1371/journal.pone.0261067 (PMC8654182; doi:10.1371/journal.pone.0261067)
Supplement: S6 Fig — The expression of PI3K, Akt and mTOR in HSC-T6 cells was analysed by western blotting. ETV, entecavir; TDF, tenofovir disoproxil fumarate; PI3K, phosphoinositide 3-kinases; Akt, protein kinase B; mTOR, mammalian target of rapamycin. (DOCX) [file pone.0261067.s006.docx]

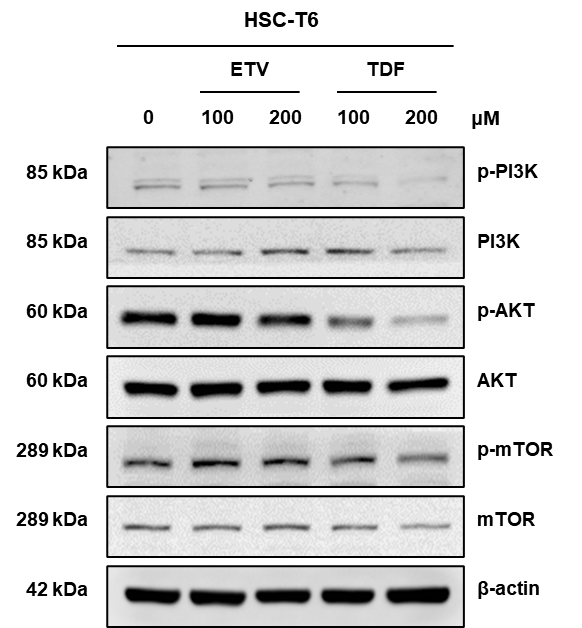


**Supplementary Fig 6. The effect of TDF on the PI3K/Akt/mTOR signalling pathway.**

The expression of PI3K, Akt and mTOR in HSC-T6 cells was analysed by western blotting. ETV, entecavir; TDF, tenofovir disoproxil fumarate; PI3K, phosphoinositide 3-kinases; Akt, protein kinase B; mTOR, mammalian target of rapamycin.
