# Supplementary material for: Tenofovir disoproxil fumarate directly ameliorates liver fibrosis by inducing hepatic stellate cell apoptosis via downregulation of PI3K/Akt/mTOR signaling pathway
Source: PLoS One. 2021 Dec 8;16(12):e0261067. doi: 10.1371/journal.pone.0261067 (PMC8654182; doi:10.1371/journal.pone.0261067)
Supplement: S7 Fig — (A and B) Smad or non-Smad signalling pathway proteins in LX2 cells were measured by western blotting. All data are representative of at least three independent experiments. ERK, extracellular signal-regulated kinase; ETV, entecavir; JNK, c-Jun N-terminal kinase; TDF, tenofovir disoproxil fumarate; TGF-β, transforming growth factor beta. (DOCX) [file pone.0261067.s007.docx]

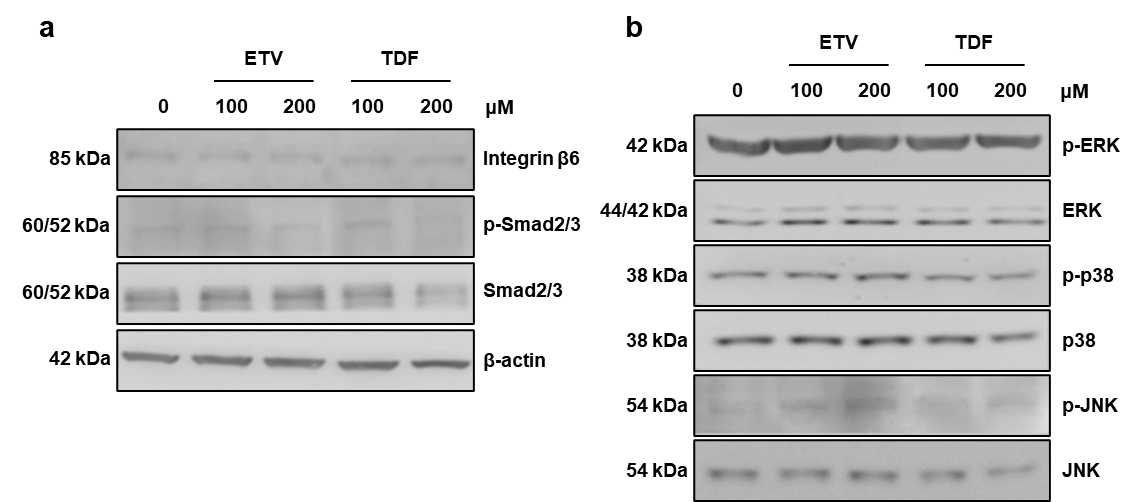


**Supplementary Fig 7. The effect of TDF on TGF-β signalling pathway components in LX2 cells.**

(A and B) Smad or non-Smad signalling pathway proteins in LX2 cells were measured by western blotting. All data are representative of at least three independent experiments. ERK, extracellular signal-regulated kinase; ETV, entecavir; JNK, c-Jun N-terminal kinase; TDF, tenofovir disoproxil fumarate; TGF-β, transforming growth factor beta.
