# Supplementary figures and images for: Tenofovir disoproxil fumarate directly ameliorates liver fibrosis by inducing hepatic stellate cell apoptosis via downregulation of PI3K/Akt/mTOR signaling pathway
Source: PLoS One. 2021 Dec 8;16(12):e0261067. doi: 10.1371/journal.pone.0261067 (PMC8654182; doi:10.1371/journal.pone.0261067)

**Figure 1**


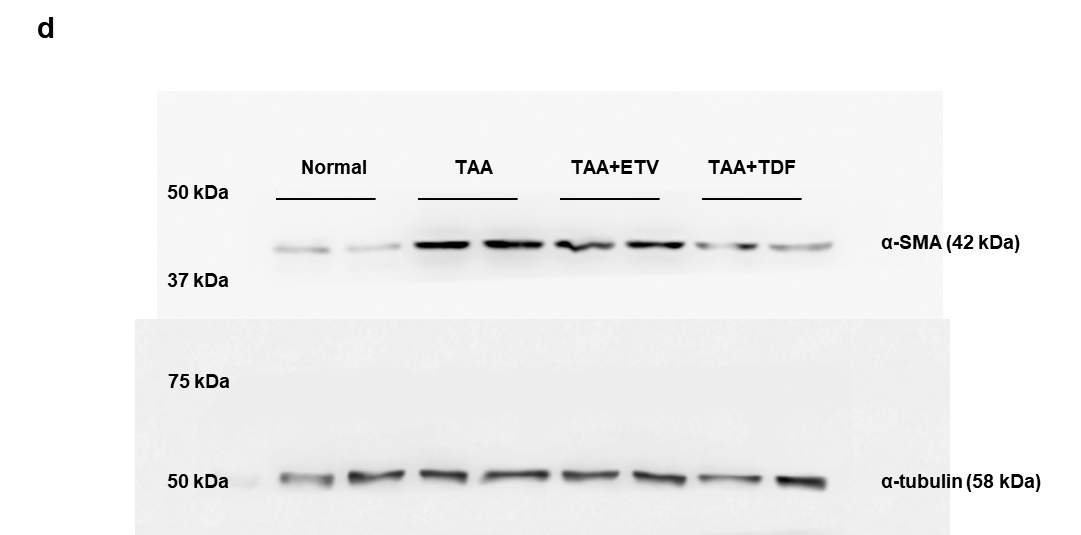


**Figure 4**


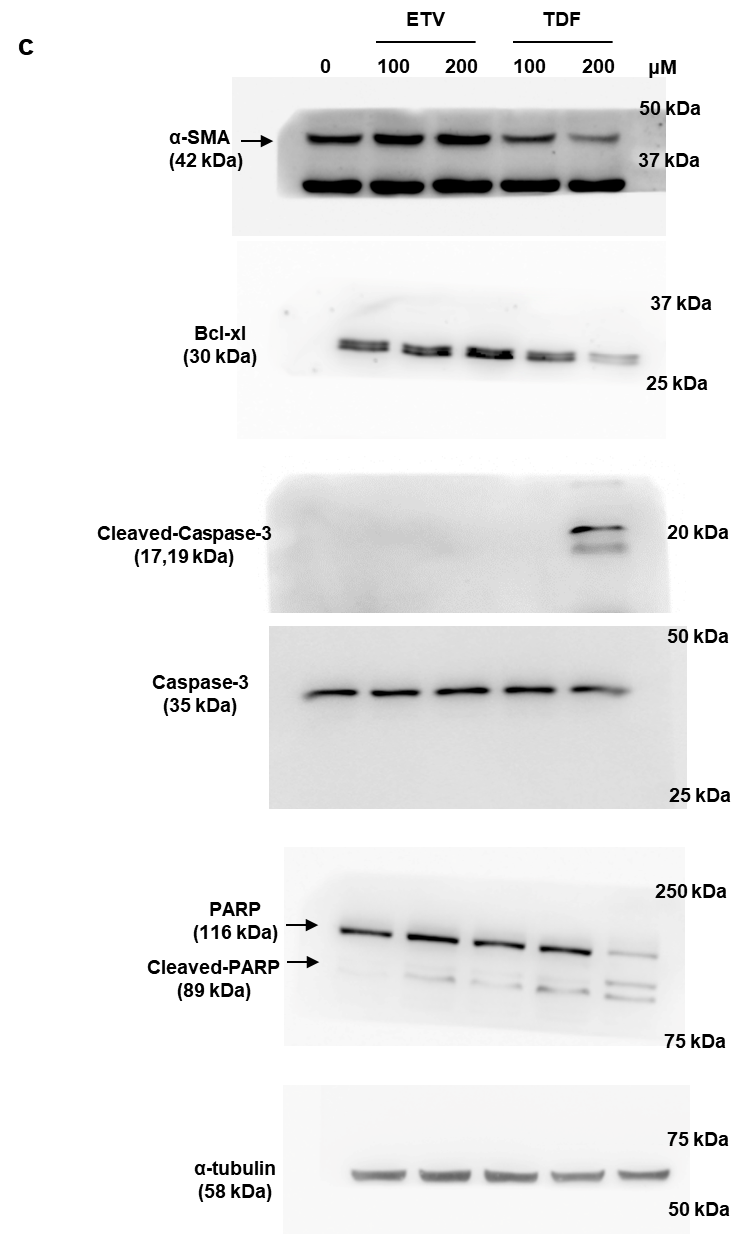


**Figure 5**


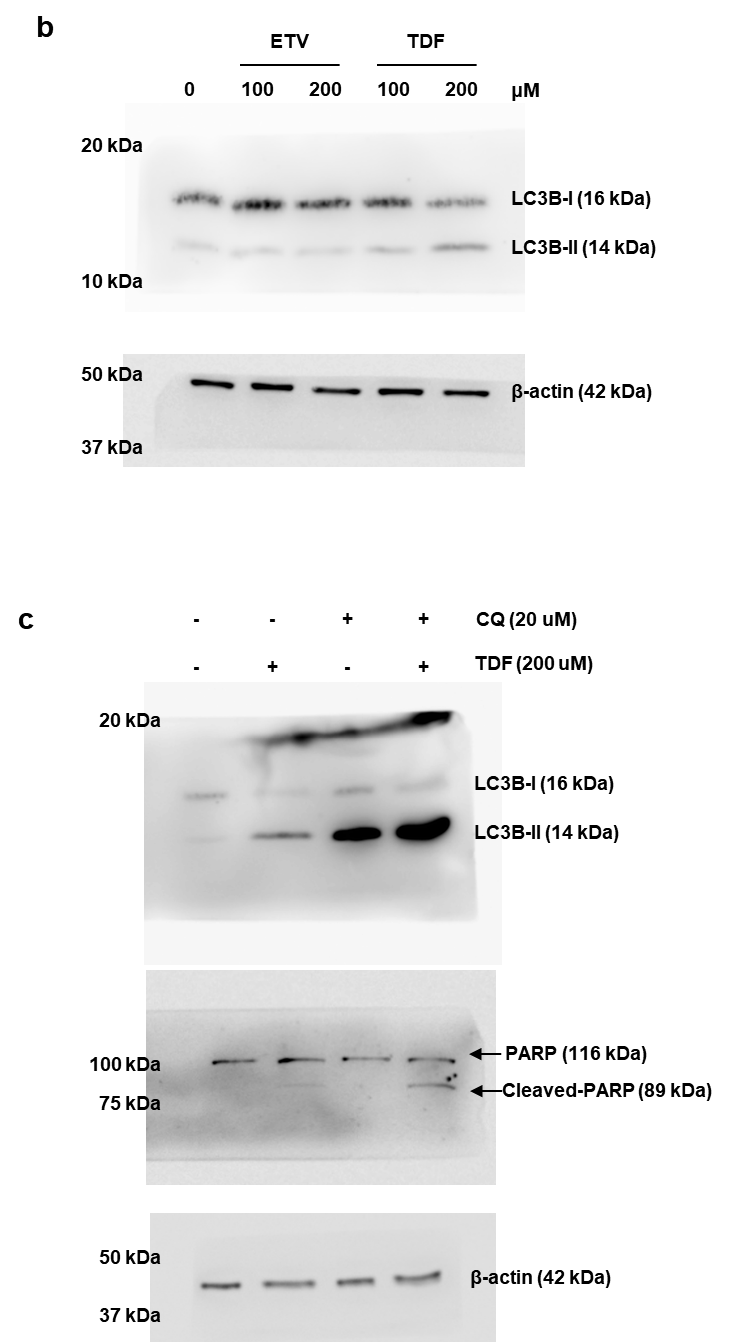


**Figure 6**


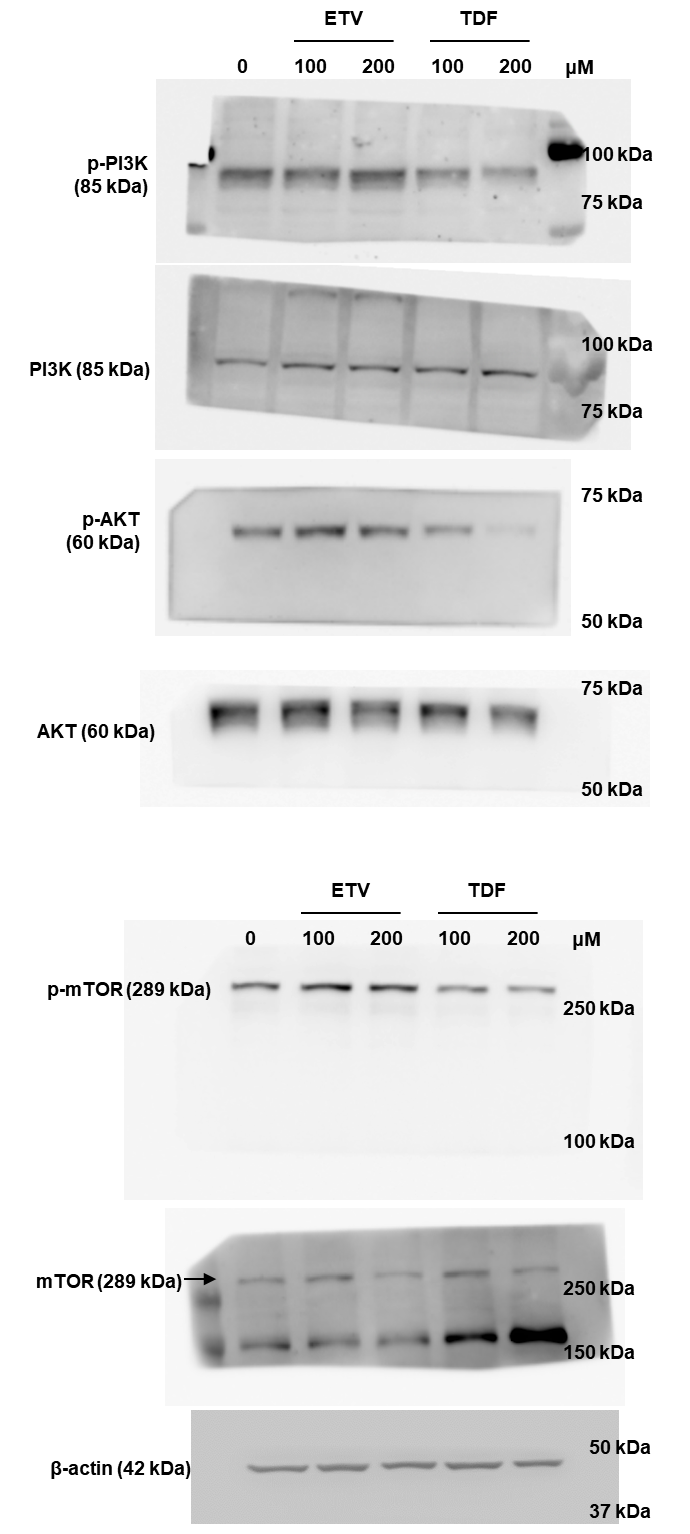

Supplement: S8 Fig — (DOCX) [file pone.0261067.s008.docx]
