# Supplementary material for: Tenofovir disoproxil fumarate directly ameliorates liver fibrosis by inducing hepatic stellate cell apoptosis via downregulation of PI3K/Akt/mTOR signaling pathway
Source: PLoS One. 2021 Dec 8;16(12):e0261067. doi: 10.1371/journal.pone.0261067 (PMC8654182; doi:10.1371/journal.pone.0261067)
Supplement: S1 Table — (DOCX) [file pone.0261067.s009.docx]

**Supplementary Table 1. Primary antibodies used for western blot.**

| **Name** | **Citation** | **Supplier** | **Cat no.** | **Clone no.** |
| --- | --- | --- | --- | --- |
| PARP |  | Cell Signaling | #9532 |  |
| Cleaved caspase3 |  | Cell Signaling | #9661 |  |
| Bcl-xl |  | Cell Signaling | #2762 |  |
| α-SMA |  | Sigma | A2547 |  |
| α-tubulin |  | Sigma | T5168 |  |
| LC3B |  | Cell Signaling | #3868 |  |
| p-PI3K |  | Cell Signaling | #4228 |  |
| PI3K |  | Cell Signaling | #4292 |  |
| p-AKT |  | Cell Signaling | #4060 |  |
| AKT |  | Cell Signaling | #9272 |  |
| p-mTOR |  | Cell Signaling | #2971 |  |
| mTOR |  | Cell Signaling | #2972 |  |
| Integrin β6 |  | Sigma-Aldrich | 407317 |  |
| p-Smad2/3 |  | Cell Signaling | #8828 |  |
| Smad2/3 |  | Cell Signaling | #8685 |  |
| p-ERK |  | Santa Cruz | Sc7383 |  |
| ERK |  | Santa Cruz | Sc-93 |  |
| p-p38 |  | Cell Signaling | #9211 |  |
| P38 |  | Cell Signaling | #8690 |  |
| p-JNK |  | Cell Signaling | #4668 |  |
| JNK |  | Santa Cruz | Sc-137018 |  |
| β-actin |  | Sigma-Aldrich | A1978 |  |
