# Supplementary material for: Tenofovir disoproxil fumarate directly ameliorates liver fibrosis by inducing hepatic stellate cell apoptosis via downregulation of PI3K/Akt/mTOR signaling pathway
Source: PLoS One. 2021 Dec 8;16(12):e0261067. doi: 10.1371/journal.pone.0261067 (PMC8654182; doi:10.1371/journal.pone.0261067)
Supplement: S1 File — (DOCX) [file pone.0261067.s010.docx]

**Supplementary methods**

***Cell viability and apoptosis assays***

Cell viability was evaluated by MTT assays using thiazolyl blue tetrazolium bromide (Sigma-Aldrich, St Louis, MO, USA). Briefly, LX2 and HSC-T6 cells (1 x 10^4^ cells/well) were seeded in each well of 96-well plates. After 24 h, various concentrations of antiviral drugs were added to each well and incubated for 24 h. Then, 20 μl of 2 mg/mL MTT solution in DMEM was added to each well and incubated at 37 ℃ for 2 h. After removal of the medium containing MTT, 100 μl of DMSO was added to dissolve the formazan crystals formed by live cells. The optical density was measured at 540 nm with a SpectraMax 250 microplate reader (Molecular Devices, Sunnyvale, CA, USA).

To assess cellular apoptosis, antiviral drug-treated LX2 and HSC-T6 cells were stained with Annexin V-FITC/PI apoptosis detection kits (BD Biosciences, San Jose, CA, USA) according to the manufacturer’s instructions and then analysed using a FACS Canto 2 flow cytometer (BD Biosciences). A TUNEL assay was used to detect and visualize apoptotic cells in LX2 cell and mouse liver tissue using an *in situ* cell death detection kit (Roche Diagnostics GmbH, Mannheim, Germany) according to the manufacturer’s protocols. The sections were stained and mounted with mounting medium containing 4,6-diamidino-2-phenylindole (DAPI; Sigma-Aldrich). Apoptotic cells were quantified by counting the number of TUNEL-positive nuclei. For each sample, the number of TUNEL-positive cells was observed under a fluorescence or confocal microscope (Zeiss, Jena, Germany).

***Quantitative real-time PCR***

Complementary DNA (cDNA) was synthesized to determine the mRNA expression of target genes using RNA isolated from mouse liver tissue as previously described. PCR was performed using specific primers. The primer sequences were as follows (5’→3’): collagen type I alpha 1 chain (Col1a1), forward: AGA TGG CAT CCC TGG ACA and reverse: GGA CAT CTG GGA AGC AAA GT; and TIMP metallopeptidase inhibitor 1 (Timp1), forward: GCA AAG AGC TTT CTC AAA GAC C and reverse: AGG GAT AGA TAA ACA GGG AAA CAC T. TaqMan probe-based real-time PCR amplifications were performed with a Light Cycler 480 instrument (Roche Applied Science, Indianapolis, IN).

***Western blotting***

LX2 cells and liver tissues were lysed using RIPA buffer (20 mM Tris-HCl at pH 7.5, 150 mM NaCl, 1% Triton X-100, 1% sodium deoxycholate, and 0.1% SDS) containing a protease inhibitor cocktail (Roche) and phosphatase inhibitor cocktails (100×) (Sigma-Aldrich). Protein extracts were separated by 10% SDS-PAGE and transferred to nitrocellulose membranes (Schleicher & Schuell, Dassel, Germany), and the membranes were blocked in 5% skim milk in TBS (10 mM Tris-HCl at pH 7.5 and 150 mM NaCl). The membrane was incubated overnight at 4°C with primary antibodies and then incubated with horseradish peroxidase (HRP)-conjugated anti-mouse or anti-rabbit secondary antibodies. The specific protein bands were visualized with an enhanced chemiluminescent system (ECL, Amersham Pharmacia Biotech., Arlington Heights, IL, USA) according to the manufacturer's instructions. The protein band densities were determined by densitometry and normalized to α-tubulin or β-actin as a loading control. The primary antibodies are listed in Supplementary Table 1.

***Measurement of liver collagen***

To evaluate collagen deposition in liver tissues, Sirius Red staining was performed using the Picrosirius Red staining kit (Abcam, Cambridge, UK) according to the manufacturer’s instructions. Five representative fields were evaluated using ImageJ software (NIH, USA) for each mouse in all experimental groups. Collagen deposition was also quantified using a hydroxyproline assay kit (BioVision, Milpitas, CA, USA) according to the manufacturer’s protocol.

***Histological examination and immunofluorescence staining***

Liver tissues were fixed in 10% buffered formalin and embedded in paraffin for haematoxylin and eosin (H&E) staining. Additionally, liver tissues were snap-frozen in OCT-embedding compound, and 7-μm frozen sections were cut, fixed with 4% paraformaldehyde and incubated with an α-SMA antibody. Alexa Fluor 546-conjugated anti-mouse IgG (Invitrogen, Carlsbad, CA, USA) was used as a secondary antibody. DAPI was used at a dilution of 1:2000 for counterstaining. The fluorescence was measured by confocal microscopy.

For liver cell imaging, the cells were seeded in a 6-well plate at a concentration of 1 x 10^5^ cells/well in 2 ml of growth medium. After 8 h of treatment with TDF, the cells were incubated for 2 h with 75 nM LysoTracker Red (Invitrogen) at 37°C. DAPI was used for counterstaining, and the fluorescence was measured by confocal microscopy.

For transmission electron microscopy, the antiviral drug-treated cells were collected and fixed with 4% paraformaldehyde and 2.5% glutaraldehyde in 0.1 mol/L phosphate buffer (pH 7.2) at 4°C overnight. After being rinsed with 0.1 mol/L phosphate buffer 3 times for 30 minutes each, the cells were treated with 1% osmium tetroxide in 0.1 mol/L phosphate buffer for 1 h, dehydrated through a graded series of ethanol and acetone, embedded in Epon 812, and polymerized at 60°C for 3 days. Ultrathin sections (60–70 nm) were prepared using an ultramicrotome (Leica Ultracut UCT; Leica Microsystems GmbH, Wetzlar, Germany). The sections were mounted on Formvar-coated slot grids (200 mesh) and examined under a transmission electron microscope (JEM 1010; JEOL Ltd., Tokyo, Japan) operating at 60 kV. Images were recorded using a CCD digital camera (Orius SC1000; Gatan, Pleasanton, CA, USA). All experiments were repeated 3 times to ensure reproducibility.
