# Supplementary material for: Tenofovir disoproxil fumarate directly ameliorates liver fibrosis by inducing hepatic stellate cell apoptosis via downregulation of PI3K/Akt/mTOR signaling pathway
Source: PLoS One. 2021 Dec 8;16(12):e0261067. doi: 10.1371/journal.pone.0261067 (PMC8654182; doi:10.1371/journal.pone.0261067)
Supplement: S2 File — (DOCX) [file pone.0261067.s011.docx]

| Fig 1. (B) | Fig 1. (C) |
| --- | --- |
|  |  |
| Fig 1. (D) |  |
|  |  |

| Fig 2. (A) | Fig 2. (B) |
| --- | --- |
|  |  |
|  |  |
|  |  |

| Fig 3. (A) |
| --- |
|  |
| Fig 3. (B) |
|  |

| Fig 3. (D) | |
| --- | --- |
|  | |
|  |  |
| Fig 4. (A) | |
|  |  |

| Fig 5. (B) | |
| --- | --- |
|  | |
| Fig 5. (C) | |
|  |  |
| Fig 5. (D) | |
|  | |
|  |  |
|  |  |

| Fig 6. |
| --- |
|  |
|  |
|  |
